# Perception and adoption of a new agricultural technology: Evidence from a developing country

**DOI:** 10.1016/j.techsoc.2018.07.007

**Published:** 2018-11

**Authors:** Khondoker A. Mottaleb

**Affiliations:** Agricultural Economist, Socioeconomic Program, International Maize and Wheat Improvement Center (CIMMYT), Carretera Mex-Veracruz, Km. 45, El Batan, Texcoco, CP 56237, Mexico

**Keywords:** Adoption, Axial-flow-pump, Centrifugal pump, Farmer, Irrigation, Low-lift-pump, Technology, Perception, Price, Society

## Abstract

Adoption of new agricultural technologies is always at the center of policy interest in developing countries. In reality, despite the visible benefits of many of the new agricultural technologies, including machinery and management practices, farmers either do not adopt them or it takes a long time to begin the adoption process and scaling up. To enhance the provision of irrigation using surface water and to enhance irrigation efficiency, Bangladesh has been trying to introduce the axial-flow-pump (AFP) appropriate for surface water irrigation, which can lift up to 55% more water, conditional on the water head, than a conventional centrifugal pump. Despite the visible benefits of the AFP, the uptake of the AFP for irrigation is low in the targeted zone of Bangladesh. The present study demonstrates that the new technology must be modified to adapt to local demand and specifications. Most importantly, the price of the new technology must be competitive with the prices of the existing available substitute technologies to ensure a rapid uptake and scaling up of this new agricultural technology.

## Highlights

• Technology adoption is core of agricultural development.
• Despite visible benefits many technologies are not adopted by farmers.
• The study used primary data to examine why this can be the case.
• The study shows that technology must be compatible to local environment.
• Price of new technology must be competitive to the alternatives.

## Graphical abstract

**Figure:**
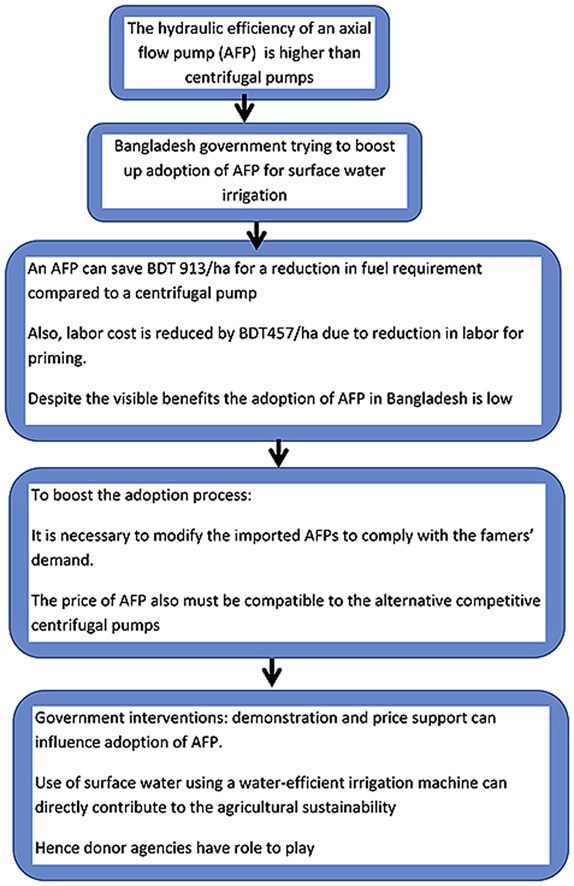

## 1 Introduction

Nearly 11% of the 7.42 billion world population is extremely poor. They are concentrated mostly in the rural areas of Southern Asia and sub-Saharan African countries, 78% of whom rely on agriculture for their livelihoods [1,2]. As the majority of the rural poor depend on agriculture for their livelihoods, agricultural growth can have paramount impacts on rural poverty alleviation. In fact, agricultural GDP growth is at least twice as effective in reducing poverty as GDP growth in other sectors [3]. It means if a 1% increase in GDP in any non-farm sector can lead to a reduction of poverty by 1%, the poverty reduction will be 2% with 1% growth in the agricultural GDP [3]. Because of its profound impacts on poverty alleviation, ensuring agricultural growth is the center of the development policies, particularly in poverty-stricken agrarian developing countries.

However, the sustainable growth of the agricultural sector critically depends on the adoption of improved, scale-appropriate and eco-friendly technologies, including new disease-resistant and climate-adjusted seeds, modern management practices, and conservation of resources using scale-appropriate new agricultural machinery. The adoption of new technology in agriculture is, therefore, at the core of agricultural growth and, thus, rural poverty alleviation. Unfortunately, the adoption of new agricultural technology, including agricultural machinery, is seldom rapid [4], as a large number of factors can affect the adoption process [[5], [6], [7], [8], [9], [10], [11]]. This is because, new agricultural technologies are often correlated with risks and uncertainties about proper application, scale appropriateness and suitability with the prevailing environment, and importantly with farmers' perceptions and expectations [3]. Examining farmers' perceptions of a new agricultural technology is, therefore, critically important to ensure the adoption and scaling up of the technologies, thereby, ensuring sustainable growth and development of the agriculture sector.

Using primary data collected from 70 sampled irrigation service providers in Bangladesh, who were given an axial-flow-pump (AFP) for free for a season under a demonstration program, and by examining the users' perceptions, the present study demonstrates the need for continuous modification of a new technology based on the requirements of the farmers. Modifications should be done at least in the initial stage of the development and deployment of a technology for the rapid adoption of a new agricultural technology. It can minimize the gap between the actual and the expected performance of a new agricultural technology, which in turn can critically influence the adoption and diffusion of that new technology. In addition, the price of a new technology must be competitive with alternative competing available technology.

The case is worth investigating for several reasons. The physical size of Bangladesh (147,570 km^2^) is almost equivalent to the US state of Georgia, yet the population of Bangladesh (158.9 million) at present is almost half of the entire US population [12]. Yet, Bangladesh is one of the few countries that has achieved rice production self-sufficiency. In the early 1970s, with a population of less than 70 million, the country faced massive food shortages and famine [13,14]. In 1970, the total cereal production in Bangladesh (rice, wheat and maize) was 16.8 million tons, which increased to 56.4 million tons by 2016 [15]. Consequently, currently with a population of nearly 160 million, the country is almost self-sufficient in food production. In 2016, with a production of 52.6 million tons of only paddy rice, Bangladesh ranked as the fourth largest paddy-rice-producing country in the world, after China, India and Indonesia [15].

The remarkable success in cereal production, and particularly in paddy production, thereby achieving rice-food production self-sufficiency, is mainly attributed to the rapid adoption of modern high-yielding varieties (HYV) along with the expansion of the ground-water-based, private-led, small-scale shallow tube well-based irrigation system [14]. However, the massive extraction of groundwater for irrigation in the entire Indo-Gangetic Plain (IGP) including Bangladesh has substantially reduced the groundwater table in Pakistan, India and Bangladesh [16]. Despite recurrent floods in Bangladesh, the ground-water level in the northwest and southwest regions has been declining by between 0.01 and 0.05 m/year [17,18]. Considering the consequences of rapidly-depleting ground-water reserves on sustainable development in the future, some studies considered the phenomenon as a major policy failure [19]. Alarmingly, in addition to the problem of the declining ground water, global climate change can also generate severe threats on sustainable agriculture in the entire IGP – the most densely-populated and intensively-cultivated region in the world. In the arid and semi-arid regions of Asia, it is estimated that the irrigation water demand will increase by 10% at the lowest for a 1 °C increase in temperature [20,21]. As an enhancement of irrigation effectiveness can reduce water requirements by nearly 50% [22], the expansion of the directly-renewable surface water-based irrigation system can be an effective remedy to the problems related to over extraction of ground water in the entire IGP including Bangladesh. In this case, the deployment of hydraulically efficient AFPs in suitable cases can be instrumental in expanding surface water-based irrigation systems.

Second, in Bangladesh, farmers generally use centrifugal pumps for irrigation. A number of carefully-controlled scientific experiments by the International Maize and Wheat Improvement Center (CIMMYT), and its national partners in Bangladesh confirm that an AFP can lift from 28% to 55% more water conditional on the water head [23]. The closer the water head, the more efficient is the AFP [23]. A rapid adoption of AFPs in suitable cases, and AFP replacement of centrifugal pumps, where suitable, therefore, can reduce the costs of production of *boro* rice. The rice production costs in Bangladesh have been spiraling over the years [24]. Currently, the *boro* rice cultivation cost in Bangladesh is USD1319/ha, of which irrigation cost is 13.4% (USD178/ha) of the total cost [25]. As the benefit-cost ratio of cultivation of *boro* rice is 0.82 [25], a reduction in the irrigation costs due to a potentially rapid adoption of AFP in suitable areas can significantly improve the current benefit-cost situation in *boro* rice cultivation in the targeted regions.

Under the initiative of CIMMYT, Bangladesh, from 2012 to 13, AFPs were made available in the southern region of Bangladesh for farmers' purchase. However, from October 2013 to September 2017, so far only 888 AFPs have been purchased by the lead farmers, who also provide irrigation services to other client farmers, in the southern region of Bangladesh, and the current land coverage using AFPs is 19,287 ha [26]. Currently in Bangladesh, there are 173.2 thousand surface water-based low-lift pumps (LLPs), 1417 thousand ground-water-based shallow-tube wells and nearly 37 thousand deep tube wells are engaged in irrigating 5.31 million hectares of land [27]. The LLPs irrigate 1.16 million hectares of land, which is nearly 22% of the total irrigated land in Bangladesh [27]. The largest number of LLPs are deployed in Chattogram Division (41,514), Sylhet Division (41,384), Khulna Division (32,741), Dhaka Division (20,581) and in Barishal Division (14,459) [27]. Currently, out of a total of 173,179 low-lift pumps in Bangladesh, 163,764 were diesel engine-based and 9415 were electric motor-based irrigation pumps [27]. Overall, a replacement of the less-efficient diesel engine-based centrifugal pumps with the more efficient AFPs can improve Bangladesh's terms of trade by reducing diesel imports, because the water-lifting capacity of an AFP is high and, consequently, the required operation time for an irrigation machine with an AFP will be less than before. Consequently the total diesel demand will be lower than before. A replacement of the diesel engine-based centrifugal pumps by AFPs can also generate positive environmental externalities by reducing emissions from the existing diesel engine-based LLPs.

Finally, the government of Bangladesh has developed and approved a master plan for agricultural development in the southern region for agricultural intensification by expanding surface water irrigation facilities [28]. Note that while the average cropping intensity of Bangladesh is 194 (i.e. a piece of land is cultivated at least 1.94 times a year), the cropping intensity in the southern region of Bangladesh (Barishal, Khulna and Patuakhali) ranges between 146 and 187 [29]. Because of the current vulnerability and potential of the southwest region, the government of the United States of America has announced a special program called Feed the Future, which is initiatives to improve the livelihoods of the poor by improving the agricultural sector [30]. In Barishal and Khulna divisions, and in Chattogram and Dhaka divisions, a sizeable amount of land is kept fallow partly due to the high establishment and operation costs for irrigation [31]. A rapid diffusion of the AFP, particularly in the southern region targeting bringing suitable fallow land under cultivation in the dry season by irrigating using AFPs, can be an efficient strategy in implementing the agricultural master plan of the government of Bangladesh.

The present study explores the factors that affect the adoption of AFPs in Bangladesh and the estimates of the expected price of an AFP. The rest of the study is organized as follows: Section 2 includes the materials and methods and elaborates the data collection process; Section 3 specifies the econometric estimation process; Section 4 presents the major findings and Section 5 presents the conclusions and policy implications.

## 2 Materials and methods: study design, study area and sampling

The International Maize and Wheat Improvement Center (CIMMYT), Bangladesh, under the Cereal Systems Initiative for South Asia – Mechanization and Irrigation (CSISA-MI) project, introduced AFPs in Bangladesh through imports from other Asian countries. Although the use of AFPs in Asia started in the 1970s, first in the Mekong delta of Vietnam [32], the AFP is a completely new technology in Bangladesh. Under a joint venture agreement with a local Bangladeshi private business organization, Rangpur Foundry Limited (RFL), and in collaboration with an international NGO, iDE (International Development Enterprise), Bangladesh, CIMMYT, Bangladesh imported and tested the performance of AFPs. A carefully-controlled scientific experiment shows that the hydraulic performance of an AFP is higher at low lift, which ranged from 28% higher at 3-m water heads to 55% higher at 2-m water heads [23]. In general, the nearer the water head, the more efficient is an AFP. The efficiency of an AFP is also influenced by the slope of an AFP: a more parallel setting of a pump provides more water lifting efficiency.

To introduce this highly-efficient irrigation pump to farmers, CIMMYT, Bangladesh organized a number of AFP-based irrigation demonstrations in the southern part of Bangladesh. Under the program, AFPs were provided for free for a season to a selected number of irrigation service providers, who were using centrifugal pumps. In addition, mechanical and technical supports and fuel subsidies were provided during demonstrations to the service providers who were selected for using AFPs under the demonstration program, keeping their centrifugal pumps idle for a season.

**Fig. 1:**
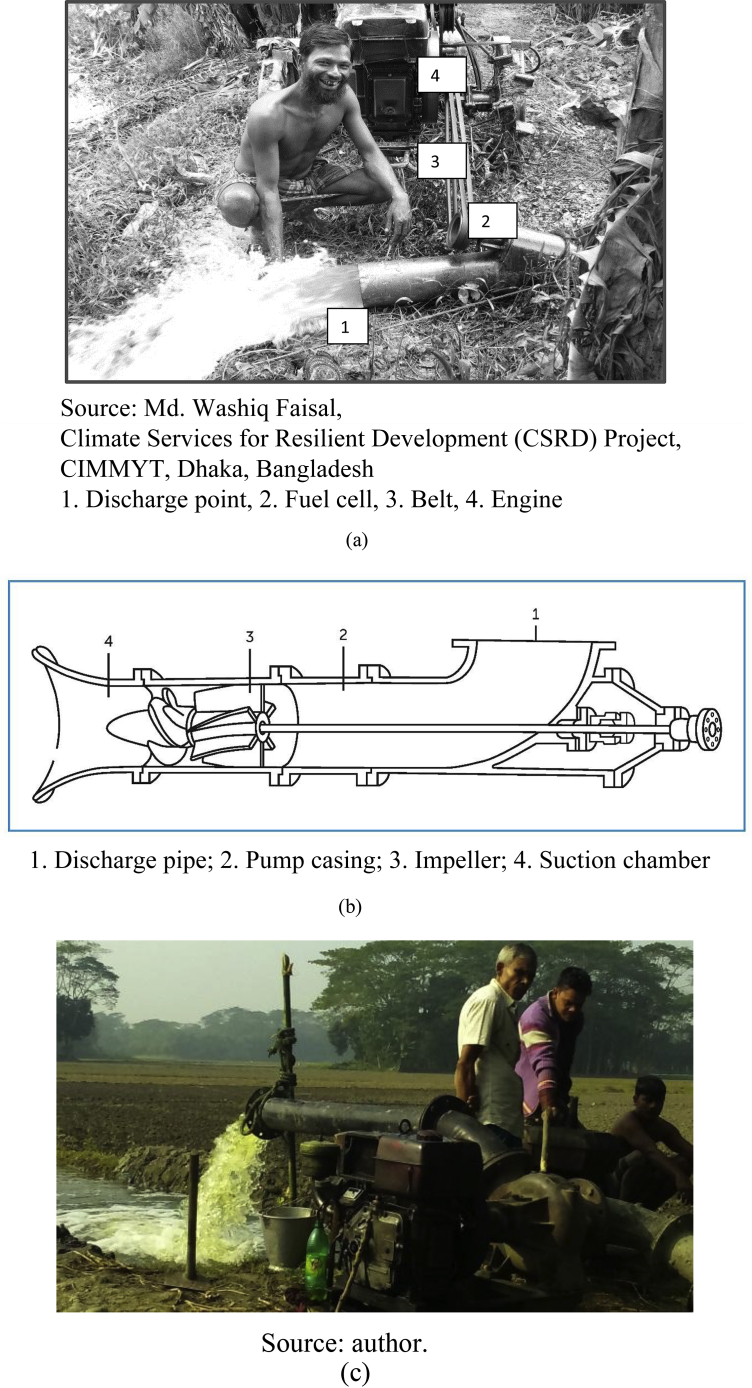
Panel (a) an axial flow pump in operation; Panel (b) inside an axial flow pump; Panel (c) a low-lift pump.

The major objectives of AFP deployment and demonstrations were to generate awareness among the irrigation service providers and to understand the perception of the service providers on AFPs, compared to the centrifugal pumps that the service providers had been using. Under the program, each selected service provider was provided with an AFP. The ultimate objective of the AFP demonstration program was to encourage irrigation service-provider farmers' to purchase AFPs. In the series of demonstrations in 2014–15, CIMMYT, Bangladesh deployed 68 AFPs in Barishal, Bhola, Barguna, Jhalokati, Patuakhali and Pirojpur districts in Barishal Division, two in Faridpur and Rajbari districts in Dhaka Division and 13 in Narail, Khulna and Satkhira districts of Khulna Division. In Barishal and Dhaka divisions, service providers used AFPs mainly for irrigating *boro* rice. In contrast, AFPs in Khulna Division were mainly used for water conveyance between/among hatcheries and ponds or aquaculture (see Fig. 1).

**Fig. 2:**
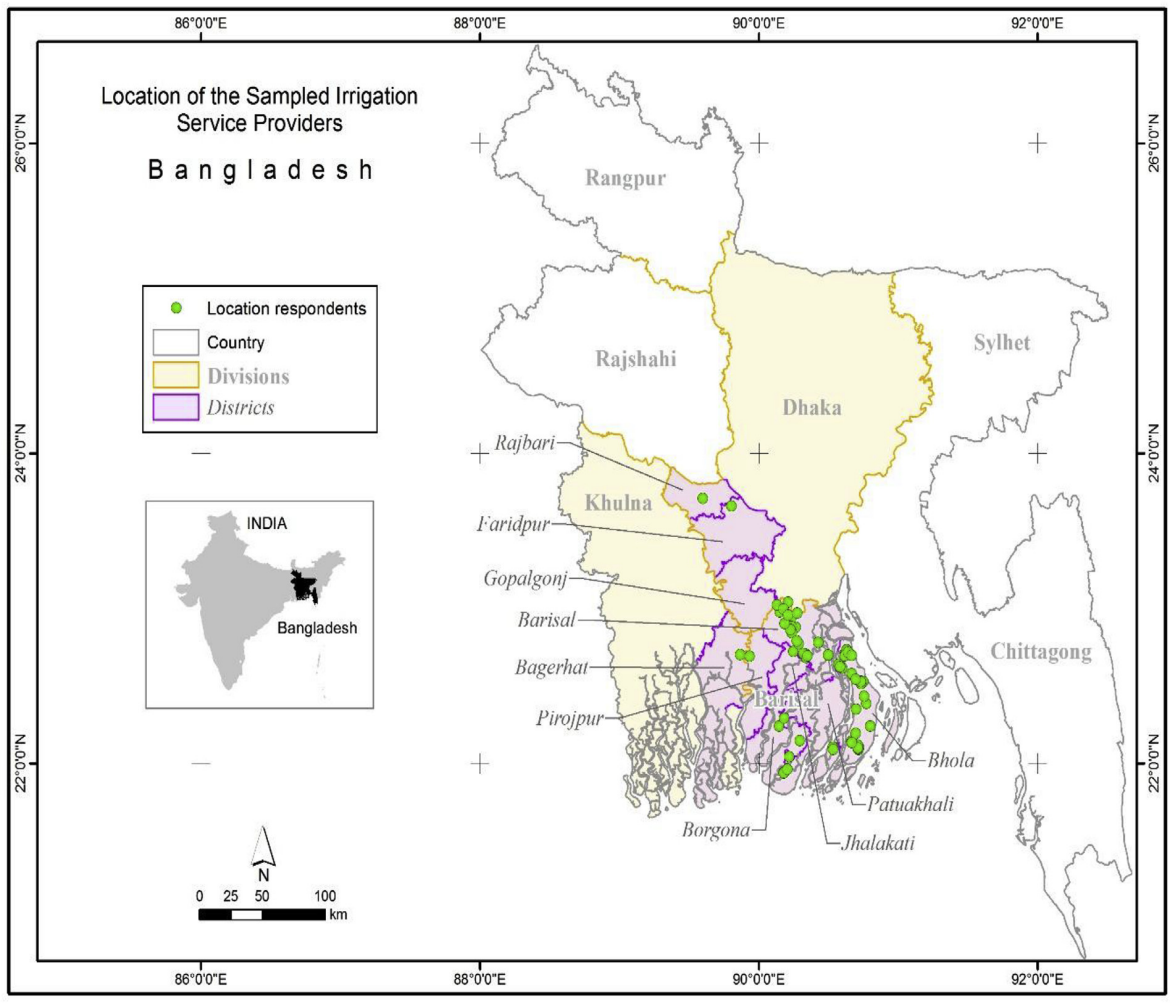
Location of the sampled irrigation service providers using AFPs in the sampled eight districts of Bangladesh.

At the end of the *boro* season in June 2015, using a standard questionnaire, the perceptions of service providers, including their willingness to purchase an AFP, were collected. As the use of AFPs in the Khulna regions was other than for crop irrigation, in this study, we have not included the perception of the AFP users in Khulna Division. The present study relies on information collected from 70 irrigation service providers located in Barishal, Bhola, Barguna, Jhalokati, Patuakhali and Pirojpur districts of Barishal Division, and Faridpur and Rajbari districts of Dhaka Division (Fig. 2). In the perception analysis process, the sampled service providers were asked to rank a number of attributes of an AFP compared to the centrifugal pump. For an attribute with the lowest level of satisfaction, service providers ranked a 1; for the highest level of satisfaction, they ranked a 4. The comparison of each attribute between an AFP and the centrifugal pump revealed by a sampled user was then tested applying a two sampled mean test-the *t*-test.

In addition to a simple two-by-two table revealing the price of the AFP that the users were willing pay, applying a two-part estimation method, we also estimated the expected price of an AFP that an AFP user offered. The application of a two-part model estimation procedure is suggested to examine the factors that affect the decision to purchase and the price to be offered for AFP by an irrigation service provider, as it permits the censoring mechanism for the irrigation service providers for whom the demand is 0, and the price function can be estimated in a separate process. More clearly, the two-part model is a special type of mixture model, in which the zero and non-zero values (price of an AFP that the users offered, in this case) will generate different separate density functions. Particularly, for the irrigation service providers who were not willing to purchase an AFP, zeros are generally handled by developing a model only for the probability of positive outcome as follows:

(1) ∅(y>0)=Pr(y>0|Z)=F(Zδ)

where Z is a vector of variables that includes all explanatory variables, δ is the related vectors of parameters, which are to be estimated and *F* is the cumulative distribution function of the error term which is indepdently and identically distributed. For the corresponding positive outcome related to probabilties, the model can be specified as:

(2) ∅(y|y>0,Z)=g(Zϒ)

where Z is the vecor of independent variables and ϒ are the corresponding vectors of parameters to estimate and *g* is the density function for y|y > 0, where the density function is necessary to select based on the distribution of y|y > 0. The likelihood contribution of an observation can be written as:

(3) $$\emptyset \left(y\right)={\left\{1-F\left(Z\delta \right)\right\}}^{i\left(i=0\right)}X\;{\left\{F\left(Z\delta \right)\;g\left(Zϒ\right)\right\}}^{i\left(y>0\right)}$$

where *i* (.) is the indictator function. Then the log-likelihood contribution on an observation can be written as:

(4) ln{∅(y)}=i(i=0)ln{1−F(Zδ)}+i(i>0)[ln{(F(Zδ)}+ln{g(Zϒ)}]

As the δ and ϒ parameters in the log-likelihood contribution for every observation are additively separable, it is possible to estimate separate models for zeros and positives. Based on the first principle of a statistical decomposition of a joint distribution into marginal and conditional distributions, the overall mean can be written as the product of expectations from the first (Equation (1)) and the second part (Equation (2)) of the model as follows:

(5) E(y|Z)=Pr(y>0|Z)XE(y|y>0,Z)

The detailed specificant of the two-part model can be seen in Belotti et al. (2015) [33]. Empirically, to identify the factors that affect the willingness to purchase and the price offered for an improved surface water irrigation pump, the AFP, in the southern regions of Bangladesh, the vector of variable Z includes:

- age of the sampled service provider;
- an education dummy that assumes a value of 1 if a sampled service provider has formal education of at least five years, or 0, otherwise;
- years of schooling of the spouse;
- a major occupation dummy that assumes a value of 1 if agriculture was the major occupation of the irrigation service provider, or 0 otherwise;
- number of earning family members;
- a two-wheeled tractor dummy, that assumes a value of 1 if a sampled service provider used an AFP as an attachement to his two-wheeled tractor, or 0, otherwise;
- a crop dummy that assumes a value of 1, if the AFP was used to irrigate boro rice, and 0 otherwise;
- land owned by the sampled irrigation service provider (ha);
- discharge diameter (inches) of the AFP that the sampled service provider used;
- length (feet) of the AFP that the sampled service provider used; and
- a dummy if the sampled service provider was in Bhola District, and 0 otherwise.

## 3 Descriptive findings: information on service providers and their perceptions on the AFP

Out of 70 sampled AFPs deployed for demonstrations in the 2014–15 *boro* season, 25 AFPs were deployed in Barishal District, 31 in Bhola District, three in Barguna District, six in Patuakhali District, two in Pirojpur District and one each in Jhalokati, Faridpur and Rajbari districts (Fig. 2). The AFPs under demonstration were different in length, and diameter (Table 1).

**Table 1:** Type of AFP deployed by the price of an AFP (after subsidy) and the length of the pump.

Length (feet)	Diameter (inch)	Price per AFP							Total
		9700	11000	11500	12000	12500	12700	14500	
14	4″								1
	5″								
	6″			1					
16	4″								20
	5″		6						
	6″				14				
18	4″								2
	5″								
	6″					2			
20	4″	12							47
	5″						18		
	6″							17	
Total		12	6	1	14	2	18	17	70

Out 70 AFPs deployed for demonstration, 47 were 20 feet long, 20 were 16 feet long, two were 18 feet and one was 14 feet long. Usually the price of an AFP is strictly fixed, based on the length and discharge diameter of the machine. In the initial years, to boost the adoption of AFPs, a subsidy of Bangladesh taka^1^ (BDT) 8000 was provided per AFP through the sales agents. The subsidy was independent of length and the discharge diameter. The actual prices of AFPs are, therefore, BDT 8000 plus the prices shown in Table 1. The long AFP with the wider discharge diameter was in high demand, as it can better serve *boro* rice irrigation that requires more water than any other winter crop in Bangladesh. The lowest priced AFP used for demonstration was 20 feet long with a four-inch discharge diameter, the price of which was BDT 9700 excluding the subsidy. Twelve of these pumps were deployed for demonstration in the 2014–15 *boro* season (Table 1). In contrast, the costliest AFP was 20 feet long with a six-inch discharge diameter, the price of which was BDT 14,500 per pump excluding the subsidy. Eighteen of these pumps were deployed for demonstration (Table 1). The AFPs under demonstration were mainly imported from Thailand and Vietnam, and sold in Bangladesh under five brand names: Bell, Parrot, RFL, Two-birds and Whale brands. The RFL brand of AFP is the AFP imported by the RFL Company, and the company used their own brand name for marketing AFPs in Bangladesh.

The basic human capital and demographic information of the sampled service providers selected to conduct demonstration irrigation using an AFP in the 2014–15 *boro* season in Bangladesh are presented in Table 2, based on whether or not a service provider was willing to purchase an AFP. A total of 55 irrigation service providers, out of 70 expressed their willingness to purchase an AFP, and offered BDT 12,310 on average as the price of an AFP (Table 2). Out of 70 sampled service providers, 56 of them were from Barishal and Bhola districts, which was 80% of the total sampled service providers under AFP demonstration and the other 14 (20%) were from other districts, such as Rajbari, and Faridpur. Among the sampled service providers of the Barishal districts, 38.2% of them expressed their willingness to purchase an AFP, and it was 43.6% in Bhola District.

**Table 2:** Information on human and physical capital of the sampled service providers.

Characteristics	All	Expressed interest to purchase AFP after use		a-b Mean difference, t-statistic and the level of significance
		Yes (a)	No (b)	
No. of service providers	70	55	15	
Average price willing to pay (BDT)	9672.86 (5278.31)	12310.91 (1587.88)		
% Service provider from Bhola District	44.29 (50.03)	43.64 (50.05)	46.67 (51.64)	
% Service provider from Barishal District	35.71 (48.26)	38.18 (49.03)	26.67 (45.77)	
% Service provider from other than Bhola and Barishal districts	20.0 (40.2)	18.2 (38.9)	26.7 (45.8)	
Land area (ha) provided irrigation services in 2014-15 *boro* season	9.76 (5.16)	10.3 (4.65)	7.80 (6.54)	2.49* (1.67)
No. of client farmers in 2014-15 *boro* season	24 (16)	26 (16)	20 (12)	6* (1.45)
Age, service provider	39.20 (9.30)	39.49 (9.28)	38.13 (9.63))	1.36 (0.50)
Years of schooling, service provider	6.64 (3.75)	6.51 (3.81)	7.13 (3.62)	−0.62 (−0.57)
% Service provider with non-farm sector as the major occupation	17.14 (37.96)	10.9 (8.34)	40.0 (50.7)	−29.09*** (2.75)
Years of experience as an irrigation service provider	11.31 (8.00)	10.89 (8.35)	12.90 (6.60)	−1.98 (−0.85)
Years of schooling, spouse	6.64 (3.63)	6.40 (3.70)	7.53 (3.31)	−1.13 (−1.07)
No. of earners in the family	1.64 (0.92)	1.53 (0.84)	2.07 (1.10)	-.054** (2.07)
% Used two-wheeled tractor to run AFP	25.71 (44.02)	30.91 (46.64)	6.67 (25.82)	24.24** (1.93)
% Irrigated only *boro* rice	72.86 (44.79)	74.55 (43.96)	66.67 (48.80)	7.87 (0.60)
Discharge diameter of the pump (inches)	5.31 (0.75)	5.33 (74.67)	5.27 (79.88)	0.06 (0.27)
Pump length (feet)	18.71 (1.90)	18.65 (1.93)	18.93 (1.83)	0.28 (0.50)

Notes: Differences = Mean (a) – Mean (b). H0: Diff = 0, H1: Diff ≠ 0 (two sided *t*-test). Values in parentheses are t-values. *Significant at the 10% level; ** significant at the 5% level and ***significant at the 1% level.

On average, a sampled irrigation service provider was 39.2 years old at the time of the survey, with 6.6 years of schooling, with 11 years of experience in the irrigation service business and with a spouse with 6.6 years of schooling (Table 2). Of the sampled service providers, 17% had their main occupation in the non-farm sector. The statistical differences in the mean (t-value) shows that the sampled service providers who were into non-farm sectors were less willing to purchase an AFP compared to other service providers, and the mean difference is statistically significant at the 1% level. On average, a sampled service provider was equipped with nearly two earning family members.

It is possible to run an AFP as an attachment to a two-wheeled tractor, which is popularly used in Bangladesh for land tilling [34]. A total of 26% of the sampled service providers used two-wheeled tractors for running their AFPs (Table 2). The sampled service providers, who used two-wheeled tractors to run the AFPs, are more willing to purchase an AFP. Of the sampled service providers, 73% used an AFP for irrigating *boro* rice and the rest irrigated wheat, water melon and other crops. The average discharge diameter of all AFPs in the demonstration was 5.3 inches with a length of 18.7 feet. The length and discharge diameter were not the decisive factors in expressing a willingness to purchase an AFP.

The comparative perceptions of a sampled service provider on the AFP they used in the 2014–15 season for irrigation and on the centrifugal pump they generally use for irrigation, are summarized in Table 3. In general, the sampled service providers have highly ranked the water lifting capacity, and the fuel and labor cost-saving attributes of an AFP compared to the centrifugal pump (Table 3). For an AFP, as the impeller is submerged under water, the priming (initial water to be filled in up to the suction point from the water head) is not required. In contrast, for a centrifugal pump, priming is a necessary requirement. Therefore, for an AFP, it is possible to save some labor cost as there is no need of priming, what is ranked as a most positive attribute of an AFP by the users (Table 3).

**Table 3:** Ranking by the sampled service providers on the selected attributes of an AFP and centrifugal pump (1 = not satisfied at all, 4 = maximum satisfaction).

Attributes of the machine	All sampled service provider		Ranking on attributes based on whether or not will purchase an AFP			
			On AFP		On centrifugal pump	
	AFP	Centrifugal pump	Yes	No	Yes	No
Water lifting	3.7 (0.51)	2.29 (0.80)	3.7 (0.47)	3.6 (0.6)	2.3 (0.85)	2.3 (0.62)
Fuel saving	3.5 (0.70)	2.14 (0.71)	3.5 (0.69)	3.4 (0.74)	2.1 (0.73)	2.1 (0.64)
Labor saving	3.6 (0.58)	1.80 (0.71)	3.6 (0.49)	3.3 (0.82)	1.7 (0.73)	2.0 (0.65)
Priming	3.9 (0.26)	1.56 (0.83)	3.9 (0.19)	3.8 (0.41)	1.5 (0.86)	1.6 (0.74)
Availability of spare parts	3.2 (1.08)	3.09 (1.06)	3.22 (1.10)	3.2 (1.01)	3.0 (1.08)	3.3 (0.98)
Availability of mechanical service	3.2 (1.09)	3.13 (1.02)	3.3 (1.12)	2.9 (0.96)	3.1 (1.02)	3.3 (1.03)
Engine cooling system	3.0 (1.01)	3.3 (0.89)	3.1 (1.02)	2.9 (1.03)	3.3 (0.95)	3.4 (0.63)
Easiness to operate	3.6 (0.54)	2.73 (0.98)	3.7 (0.46)	3.3 (0.72)	2.7 (1.02)	2.7 (0.82)
Setting with engine and machine	3.2 (0.94)	3.2 (1.0)	3.3 (0.94)	2.8 (0.86)	3.2 (1.0)	3.3 (1.03)
Mobility of the machine	3.6 (0.66)	2.43 (0.89)	3.6 (0.71)	3.7 (0.46)	2.4 (0.93)	2.5 (0.74)
Overall satisfaction level	3.7 (0.50)	2.4 (0.79)	3.7 (0.51)	3.7 (0.49)	2.3 (0.78)	2.5 (0.83)

In terms of the availability of spare parts, and mechanical services, the sampled irrigation service providers almost equally ranked both the AFPs and centrifugal pumps. However, in the case of the engine cooling attributes, the sampled service providers ranked the centrifugal pump higher than the AFP. In Bangladesh, in many cases, irrigation service providers make a direct water line to cool down the engine attached to the pump. The facility can be more easily generated for a centrifugal pump than for an AFP. For an AFP, to generate a direct water line to cool the engine, it is necessary to make a perfect mechanical hole with the provision of a water pipe. It requires mechanical and technical skills. The respondents ranked equally the machine and engine setting attributes of an AFP and a centrifugal pump. Currently, an AFP does not have a built-in chassis. Therefore, it is somehow problematic to connect an AFP to an engine, and an imperfect setting can lead to a repeated loss in belts that connect the engine to an AFP. In contrast, almost all centrifugal pumps are set with the engine on the same chassis and are thereby easy to operate. Finally, overall the sampled service providers were more satisfied with the overall performance of the AFPs compared to the centrifugal pumps.

During our survey, we asked the service providers whether or not they realized a reduction in fuel and labor costs, and 93% of the respondents realized a reduction in fuel cost and 90% reported a reduction in labor cost. The study revealed that, on average, compared to a centrifugal pump, using an AFP can save BDT913/ha due to a reduction in fuel requirements, and BDT457/ha due to a reduction in labor requirement primarily related to priming (Table 4). Currently, there are 173,179 units of low lift pumps (surface water irrigation pumps) in Bangladesh in operation, which irrigate 1,164,603 ha of land [27]. Based on the findings (Table 4), even if it is possible to irrigate only 10% of 1,1646,03 ha of land using AFPs by replacing the relatively less-efficient centrifugal pumps, simple calculations show that it is possible to save BDT159.5 million (approximately USD1.92 million) through a reduction in diesel and labor demands in a single season.

**Table 4:** Reported reduction in fuel and wage costs from using an AFP for irrigation.

Characteristics	Average of all sampled respondents
% Reported reduced fuel cost	92.9 (25.9)
Actual value of fuel saved (BDT)	913.5 (964.1)
% Reported reduced labor costs related to priming	90.0 (30.2)
Actual value of saved labor costs (BDT)	457.2 (809.3)

Source: Survey, 2015.

**Fig. 3:**
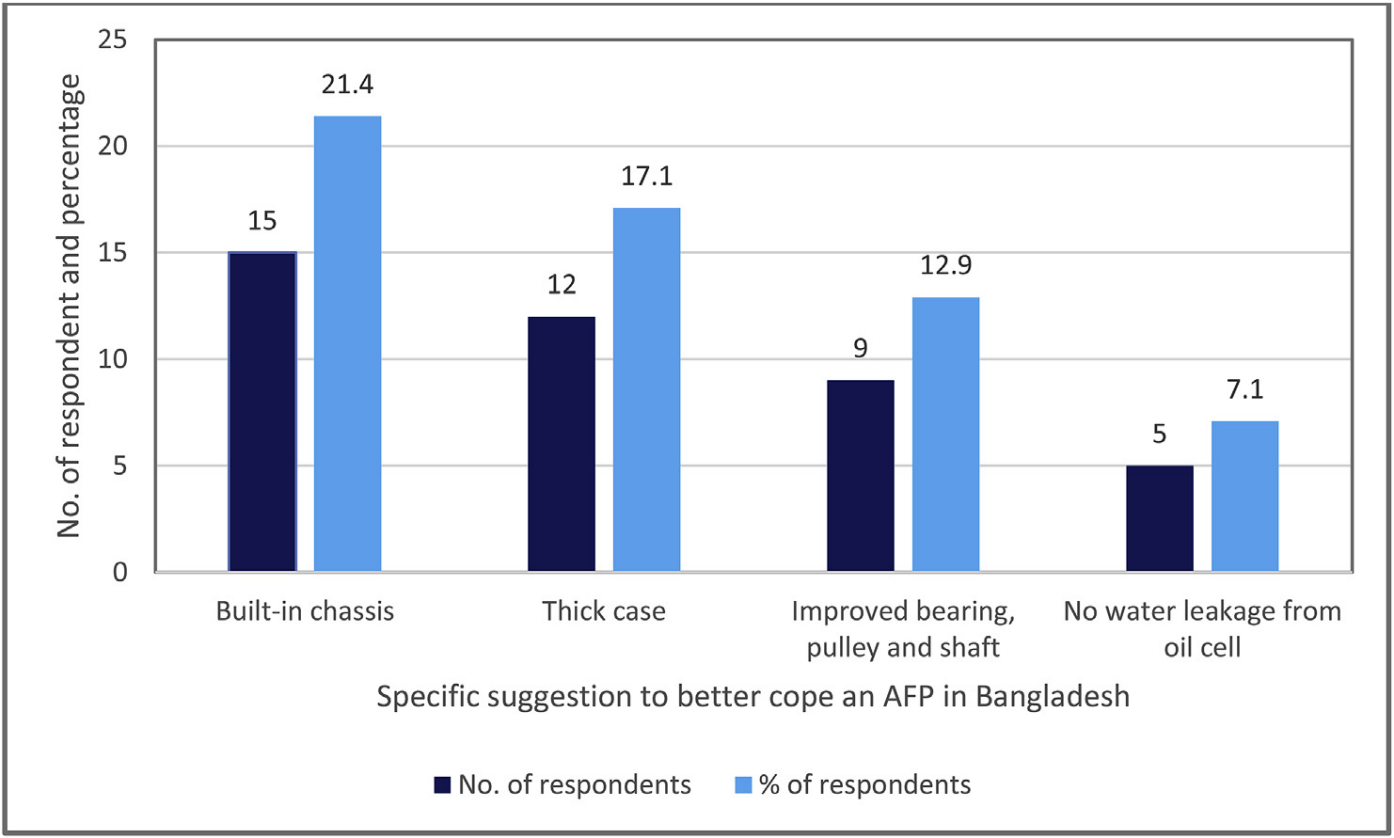
Suggestions for improving the AFP.

We asked the respondents about the attributes that are required to adjust or re-set to make the AFP user-friendly to the irrigation service providers in Bangladesh. They said that the AFP should come with a generic chassis which will be compatible with existing popular engines (Fig. 3). Of the sampled service providers, 17% suggested increasing the thickness of the pipe of the AFPs to increase the longevity by reducing the chance of denting and/or breaking the machine. After using the AFP, these service providers also mentioned that quality material for the bearings, pulley and shaft of the machine be ensured and suggested that no leakage of water from the oil cell should occur. Spilling water through the oil cell can easily damage the bearings of AFPs. Currently, the available AFPs in Bangladesh are directly imported from abroad. Suggestions of the sampled service providers indicate the need to develop and expand the local manufacturing and assembling capacity of AFPs in Bangladesh to better fit with the local demand (Fig. 3).

During our survey we informally asked the sampled respondents what the major constraint in switching from centrifugal pumps to AFPs is. They stressed two issues as major constraints to AFP adoption. First, all of the sampled service providers were using centrifugal pumps. Despite visible gains from the higher water-lifting capacity and no priming, switching from a centrifugal pump to an AFP requires a substantial amount of new investment. Second, and most importantly, the price of an AFP is comparatively much higher than a centrifugal irrigation pump, which comes as an attachment with an engine of different capacity from a 4-horsepower engine at the minimum. For an AFP, for a new irrigation service provider, it is necessary to purchase an AFP first and engine separately pairing it with the capacity of the pump. Currently, the available AFP in Bangladesh requires an 8–12 horsepower engine at the least to accrue the benefits of hydraulic efficiency. The popularity of small horsepower-engine-driven centrifugal pumps and the requirement for a high horsepower engine to run an AFP is a barrier to the rapid adoption of AFPs in Bangladesh.

The expected price that the sampled AFP users offered is calculated in Table 5. The price of an AFP is strictly determined by the length and diameter of it. Therefore, the different prices that the sampled users faced are simply because they have used AFPs of different length and diameters. Fifteen of the sampled service providers were unwilling to purchase an AFP; in contrast 55 of the sampled AFP users expressed their willingness to purchase the AFP they had used at the market price. Note that the price range presented in Table 5 are the prices after subsidy. It shows that on average a sampled AFP user offered BDT 9672.9 for an AFP.

**Table 5:** Expected price offered by the sampled users' based on the length (feet) and diameter (inches) of the pump they were supplied under the demonstration program.

Price range (BDT)	No. of farmers expressed willingness to purchase	Pump specification (length in feet/discharge diameter in inches)											
		14′			16′			18′			20′		
		4″	5″	6″	4″	5″	6″	4″	5″	6″	4″	5″	6″
0	15					1	3				3	4	4
9700–11000	14					5					9		
11500–12000	12			1			11						
12500–12700	16									2		15	
>12700	13												13
Expected price: BDT 9672.86	70			1		6	14			2	12	19	17

Source: survey, 2015.

## 4 Econometric findings

Table 2 shows that out of 70 irrigation service providers who used an AFP, 55 of them expressed an interest in purchasing an AFP (price>0) and 15 of them were not (price = 0). It allows for the application of the two-part model estimation approach to estimate the price to be offered for an AFP by an irrigation service provider.

Table 6 presents the estimated functions explaining the probability of willingness to purchase an AFP (yes = 1, no = 0), and price that a sampled irrigation service provider offered to purchase the AFP that they were using provided for free for a season by CIMMYT, Bangladesh for irrigation demonstration. In addition, the overall marginal effects of each of the variables are presented as some of the variables affected the choice function and price function differently. It shows that, on average, the level of education of a service provider positively but weakly affects the willingness to purchase an AFP (p < 0.10). A service provider with five or more years of schooling is more likely to purchase an AFP, and is willing to offer BDT 2229.2 (p < 0.10) more on average for the AFP he used compared to others. Probably, for the relatively-more educated irrigation service providers, it is easier to more accurately calculate the future stream of benefits from the current level of relatively-higher investment on an improved irrigation machine. It positively affects their decision to purchase and offer a relatively higher price for an AFP. The service providers who are engaged in agriculture full-time are more willing to purchase an AFP (p < 0.001) and, on average, they are willing to pay BDT 3866.2 more for an AFP than the other service providers. The service providers who are completely dependent on agriculture for their livelihoods are more eager to increase their income from agriculture and, therefore, are ready to invest in more efficient agricultural machinery than others. Conversely, the service providers who are dependent on the non-farm sector for their livelihoods and agriculture is only their part-time job can receive more return from investment in the non-farm sector. Therefore, this group of service providers is not interested in investing in agricultural machinery.

**Table 6:** Function estimated applying a two-part model estimation approach explaining the factors that affect the probability of purchase and the price offered for an axial flow pump by an irrigation service provider in Bangladesh.

Estimation procedures	Probit	GLM	dy/dx
Dependent variables	Will purchase an AFP (yes = 1)	Price of the pump	Marginal effects (overall)
Age, service provider	0.033	−0.001	69.41
	(0.02)	(0.00)	(54.80)
Dummy for 5 or more years of schooling (yes = 1)	0.96*	−0.0022	2229.20*
	(0.58)	(0.01)	(1360.34)
Years of schooling, spouse	−0.085	0.0011	−193.14
	(0.06)	(0.00)	(142.59)
Dummy for agriculture is the major occupation (yes = 1)	1.55***	0.024	3866.18***
	(0.55)	(0.02)	(1101.88)
No. of earners in the family	−0.59**	−0.0046	−1423.08***
	(0.23)	(0.01)	(518.15)
Provided irrigation services to the total land in 2014-15 season (ha)	0.10*	0.0012	247.56*
	(0.06)	(0.00)	(131.28)
Dummy for attaching a two-wheeled tractor to run AFP (yes = 1)	1.91**	0.015	4621.94**
	(0.79)	(0.01)	(1839.75)
Dummy for provided irrigation only to *boro* rice (yes = 1)	0.57	−0.022	1122.38
	(0.60)	(0.02)	(1489.48)
Land owned by the irrigation service provider (ha)	−0.002	0.0016***	11.26
	(0.04)	(0.00)	(98.09)
Pump's discharge diameter (inches)	−0.71**	0.18***	77.04
	(0.31)	(0.01)	(865.49)
Pump's length (feet)	−0.10	0.043***	174.08
	(0.11)	(0.00)	(271.04)
Bhola District dummy (yes = 1)	0.90**	−0.0047	2076.02**
	(0.41)	(0.01)	(1006.47)
Constant	4.41	7.68***	
	(3.05)	(0.08)	
Observations	70	55	
Linktest	Pass	Pass	
Wald chi2 (11)	25.64		
Prob > chi2	0.01		
Pseudo R2	0.34		
Log pseudolikelihood	−23.96	−572.57	
Deviance		.070	
Pearson		.070	S
AIC		21.29	
BIC		−168.24	
Expected price that a service provider is willing to pay (BDT) for an AFP		9649.84*** (485.44)	

Note: Values in parentheses are robust standard errors.*Significant at the 10% level; ** significant at the 5% level and ***significant at the 1% level.

Interestingly, the number of earners in a family is negatively associated with the willingness to purchase an AFP and, on average, an increase in the number of the earning family member by 1 reduces the offered price for an AFP by BDT 1423. Importantly, the command area that is the size of the land that an irrigation service provider served, positively and significantly affects the willingness to purchase an AFP and the overall price a service provider is willing to offer for the AFP. It shows that, on average, a 1 ha increase in the command area that a service provider served, increases the revealed price for an AFP offered by a service provider by BDT 248 (p < 0.10) probably because an AFP can lift up to 55% more water than a centrifugal pump. Therefore, it is more beneficial for a service provider who provides irrigation services to more land and more clients. On average, the sampled service providers that attached an AFP to a two-wheeled tractor to run the machine were more interested in purchasing an AFP (p < 0.05), and offered BDT4,622 more for the AFP than others. At the lowest, the engine of a two-wheeled tractor should be a 16 horsepower. The minimum requirement to run an AFP was an 8–12 horsepower engine. It means the AFP which was run as an attachment to a two-wheeled tractor with a 16-horsepower engine performs much better than others. It positively affects the willingness to purchase and to offer a higher price by the service provider. The physical capital of the service provider in terms of his own land (ha) positively affects the price offered for an AFP, but the overall marginal effect is positive but statistically insignificant. The discharge diameter of an AFP negatively and significantly affects the willingness to purchase an AFP, but is positive and significant in explaining the price a service provider offered. The overall marginal effect of the discharge diameter is insignificant. Similarly, the length of an AFP positively and significantly affected only the price of the AFP that the service provider offered.

The overall effect of the length is insignificant in deciding to purchase an AFP and in offering a price for it (Table 6). The service providers located in Bhola districts are more willing to purchase an AFP and overall they are ready pay BDT 2076 more for an AFP than the service providers in other districts. Ancillary parameters in Table 6 indicate that the model was well fit with Pseudo R^2^ 0.34 and the linktest results suggest that there was no problem with the model specification. Finally, based on the estimated function, our study shows that, on average, an irrigation service provider in Bangladesh is expected to offer BDT 9650 for an AFP. The expected price calculated in Table 5 from descriptive analysis (BDT 9672.9) and the expected price calculated from econometric analysis (BDT 9650, Table 6) are almost the same. Although the sample size is small, the similarities in the calculated expected price of an AFP indicates the robustness of our findings.

## 5 Conclusions and policy implications

Extreme poverty is widespread among the farm households in the rural areas of developing countries. The rapid proliferation of useful technologies can have profound impacts on rural poverty. The present study demonstrates the problems related to the diffusion of a new agricultural technology despite the visible gain from the adoption of it. Using primary data collected in the 2014–15 *boro* rice season from 70 irrigation service providers in a demonstration experiment of the axial flow pump (AFP) in eight districts in Bangladesh, this study examined the factors affecting the willingness to purchase an improved agricultural technology in a developing country. In the experiment process, the irrigation service providers were selected based on the fact that they were using conventional low-lift centrifugal pumps for providing irrigation services to client farmers using surface water. Under this demonstration program, an AFP was provided to the selected irrigation service providers for free to use for a season. At the end of the season, the irrigation service providers were requested to rank a number of attributes of the AFP they used in comparison to the centrifugal pump that they were using previously.

The findings of the present study confirmed the claim that, in general, AFPs are more efficient in lifting water than the centrifugal pumps [23]. In addition, our study demonstrates that, by using an AFP instead of a centrifugal pump, a surface water-based irrigation service provider in Bangladesh can save at least BDT913/ha due to a reduction in fuel requirements as an AFP can lift more water than a centrifugal pump and, thus, requires less machine time for irrigation of the same amount of land which was irrigated the previous year using a centrifugal pump. However, despite the visible benefits of an AFP over centrifugal pumps, the uptake of the machine is low. From October 2013 to September 2018, only 888 units of AFP were sold. However, a rapid scaling up of the AFP, where its use is feasible particularly in the southern region of Bangladesh where surface water is abundant, can reduce irrigation costs and therefore the overall crop production costs significantly.

Based on the findings, the present study suggests conducting more demonstrations and awareness programs of AFP-based irrigation in the areas where there is a high potential for the expansion of irrigation using surface water, particularly, in Chattogram, Khulna, Sylhet and Barishal divisions, where surface water irrigation is prominent. Relatively well-educated irrigation service providers and service providers who are owners of two-wheeled tractors can be targeted, particularly for the rapid diffusion of the AFP in Bangladesh. In addition, based on the requirements, AFPs in Bangladesh should be adjusted to the local demand. For this, the government can provide the necessary technical support to establish local assembling units and workshops. The Government of Bangladesh also may assist in the production of AFP locally. Finally, the price of the AFPs must be competitive with the existing centrifugal pumps. Our study shows that around BDT 10,000 might be a reasonable price for an AFP on average, but this is still much higher than the price of a centrifugal irrigation pump.

The policy implications from the present study can be generalized to other agricultural technologies in developing countries. This study shows that it is necessary to make the new technology compatible with local demand and the environment, and importantly the price of the new technology must be competitive with the existing alternatives. Although it is always assumed that the market mechanism can influence the adoption and scaling up of a technology as farmers are rational, in many cases initial support in the form of subsidies and technical supports can facilitate the scaling up process of a useful technology. The present study, therefore urges the international donor agencies together with the national government to support the scaling up and the adoption of AFPs in Bangladesh, and particularly to strongly support the diffusion of useful agricultural technologies in poverty-stricken developing countries.

Note that this study is based on information collected from 70 sampled AFP users in Bangladesh. In addition, sampled respondents were selected only from Barisal and Dhaka Divisions, although there is a high potential for the expansion of AFP-based surface water-based irrigation systems in Chattogram and Sylhet divisions. Considering these factors as limitations of the present study, future research endeavors should expand the AFP-based irrigation demonstration program to all potential areas of Bangladesh.

## Appendix A Supplementary data

Data Profile Data Profile

data.zip data.zip

## Footnotes

1 Currently USD 1 = BDT 83 (approximately).
